# CD4 Dynamics over a 15 Year-Period among HIV Controllers Enrolled in the ANRS French Observatory

**DOI:** 10.1371/journal.pone.0018726

**Published:** 2011-04-21

**Authors:** Faroudy Boufassa, Asier Saez-Cirion, Jérome Lechenadec, David Zucman, Véronique Avettand-Fenoel, Alain Venet, Christine Rouzioux, Jean-François Delfraissy, Olivier Lambotte, Laurence Meyer, for the ANRS EP36 HIV Controllers Study Group

**Affiliations:** 1 Inserm, CESP Centre for Research in Epidemiology and Population Health, U1018, Epidemiology of HIV and STI Team, Le Kremlin-Bicêtre, France; 2 Université Paris-Sud, UMRS 1018, Le Kremlin Bicêtre, France; 3 Institut Pasteur, Unité de Régulation des Infections Rétrovirales, Paris, France; 4 AP-HP, Service de Médecine Interne, Hôpital Foch, Suresnes, France; 5 Université Paris-Descartes EA3620, AP-HP, Hôpital Necker, Paris, France; 6 INSERM U1012, Université Paris-Sud, Le Kremlin Bicêtre, France; 7 AP-HP, Service de Médecine Interne, Hôpital de Bicêtre, Le Kremlin-Bicêtre, France; 8 AP-HP, Service de Santé Publique, Hôpital de Bicêtre, le Kremlin Bicêtre, France; University of Toronto, Canada

## Abstract

**Background:**

There are few large published studies of HIV controllers with long-term undetectable viral load (VL). We describe the characteristics and outcomes of 81 French HIV controllers.

**Methods and Results:**

HIV controllers were defined as asymptomatic, antiretroviral-naïve persons infected ≥10 years previously, with HIV-RNA <400 copies/mL in >90% of plasma samples. All available CD4 and VL values were collected at enrolment. Mixed-effect linear models were used to analyze CD4 cell count slopes since diagnosis. HIV controllers represented 0.31% of all patients managed in French hospitals. Patients infected through intravenous drug use were overrepresented (31%) and homosexual men were underrepresented (26% of men) relative to the ANRS SEROCO cohort of subjects diagnosed during the same period. HIV controllers whose VL values were always below the detection limit of the assays were compared with those who had rare “blips” (<50% of VL values above the detection limit) or frequent blips (>50% of VL values above the detection limit). Estimated CD4 cell counts at HIV diagnosis were similar in the three groups. CD4 cell counts remained stable after HIV diagnosis in the “no blip” group, while they fell significantly in the two other groups (−0.26√CD4 and −0.28√CD4/mm^3^/year in the rare and frequent blip groups, respectively). No clinical, immunological or virological progression was observed in the no blip group, while 3 immunological and/or virological events and 4 cancers were observed in the blip subgroups.

**Conclusions:**

Viral blips in HIV controllers are associated with a significant decline in CD4 T cells and may be associated with an increased risk of pathological events, possibly owing to chronic inflammation/immune activation.

## Introduction

So-called HIV controllers (HIC) are individuals in whom HIV-1 remains undetectable without antiretroviral treatment, regardless of the CD4 cell count [1]–[5]. These patients differ from long-term non progressors (LTNP) [6]–[10], who are defined as having high and stable CD4 cell counts (>500 or 600/mm^3^). Seven to eight percent of untreated HIV-1 seroconverters have undetectable viral load on two consecutive occasions during the first years following infection [11], [12]. HIV controllers, who have durably undetectable viral load, are rarer, representing less than 1% of routinely monitored patients [13]–[14]. A better understanding of the mechanisms responsible for spontaneous sustained HIV control could assist with the development of vaccines and new therapies. Most studies of HIV controllers have focused on pathophysiological aspects, while few data are available on clinical and sociodemographic characteristics or long-term CD4 cell count dynamics after HIV diagnosis [15]. We describe the epidemiologic characteristics and disease outcomes of 81 HIV controllers recruited in 2006–2007 by the ANRS HIV Controllers French National Observatory.

## Methods

With their written informed consent, HIV controllers identified among HIV-1-infected patients managed in 35 clinical centers in France were enrolled in the ANRS EP36 national program in 2006–2007. They had to have had untreated asymptomatic HIV-1 infection for more than 10 years and plasma HIV-1 RNA levels below 400 copies/ml in more than 90% of samples tested since diagnosis. All previous CD4 T cell counts and plasma HIV-1 RNA values were collected at enrolment, as well as demographic, epidemiological and clinical data. CD4 cell counts were routinely determined by flow cytometry using standard procedures. Plasma HIV-RNA was measured with the Cobas Amplicor HIV Monitor in 69% of samples [Roche Molecular Systems, Neuilly sur Seine, France; detection limit 500 to 20 copies/mL]; the Quantiplex HIV-RNA (bDNA) assay [Bayer Diagnostics, Puteaux, France; detection limit 10 000 to 40 copies/mL] in 27% of samples; and other techniques in 4% of samples [detection limits 500 to 40 copies/mL]. Whole-blood samples were also cryopreserved at enrolment, for HIV DNA assay by a centralized laboratory using the ANRS real-time PCR assay [16]. Samples with very low values were tested in quadruplicate by PCR in order to obtain numerical data. Results were expressed as log_10_ copies per million PBMC.

The patients were divided into three groups on the basis of their viral load values since HIV diagnosis: 1) a “no blip” group (n = 30) with plasma HIV-RNA always below the detection limit (including in assays with a sensitivity of 50 copies/ml in recent years); 2) a “rare blips” group (n = 39), in which fewer than 50% of HIV-RNA values were above the detection limit; and 3) a “frequent blips” group (n = 12) in which more than 50% of HIV-RNA values were above the detection limit. In keeping with the definition of HIV controllers, at least 90% of viral load values in these latter two groups were nonetheless below 400 copies/mL.

Continuous variables were expressed as the median and inter-quartile range [IQR] or range, and qualitative variables as the frequency (n) and percentage (%). Continuous variables were compared with the Kruskall Wallis test and qualitative variables with the Pearson chi-square test or Fisher's exact test. Univariate and multivariate (after adjustment for sex, age and ethnicity) mixed-effect linear models were used to estimate changes in CD4 cell counts over time. This model was used in order to take into account the fact that the subjects had repeated CD4 measurements. Square root transformation of CD4 cell counts was used to fulfill the model assumptions. The SAS (Version 9.1, 2003; SAS Institute Inc., Cary, NC, USA) and STATA programs (Version 11.0, 2009; Stata Corp., College Station, Texas) were used for statistical analysis.

## Results

### Characteristics of HIV controllers

Among 34 317 patients managed in 35 French clinical centers, 106 HIV controllers were identified (0.31%; 95%CI [0.25%–0.37%]) and 81 of them agreed to participate in this study. Thirty-five (43%) of these 81 patients were women, 68 (84%) were Caucasian, 85% defined themselves as heterosexuals (100% of women and 74% of men), and 26% of the men defined themselves as homo-bisexual. Median age at HIV diagnosis was 29 years [range: 0.8–64] and median age at enrollment in this study was 45 years [range: 19–78]. HIV infection was attributed to sexual intercourse in 42 cases (52%), intravenous drug use in 25 cases (31%), blood transfusion in 3 cases; exposure to blood products for hemophilia in 3 cases; and mother-child transmission in 1 case; the route of infection was unknown in 7 cases. The median year of HIV diagnosis was 1989 [IQR: 1987–1994], and the median time from diagnosis to enrollment in the HIV controllers study was 17 years [IQR: 13–19].

These patients' baseline characteristics were then compared with those of the patients enrolled in the ANRS SEROCO cohort [17], [18], consisting of HIV-infected subjects diagnosed during the same calendar period and still alive in 2007 (table 1). The proportion of women tended to be higher among HIV controllers than in the SEROCO cohort (p = 0.06) but this trend disappeared after adjustment for ethnicity. Among men, the proportion of homo-bisexuals was much lower among HIV controllers than in the SEROCO cohort (26.1% vs. 54.3%; p<0.001). The proportion of intravenous drug users was higher among HIV controllers than in the SEROCO cohort. These last two differences were also noted in the Caucasian subpopulations. CD4 cell counts at HIV diagnosis were higher in HIV controllers than in the SEROCO cohort (median 795 vs. 520 CD4/mm^3^, p<0.001) and percentage of undetectable viral load observed on the first available load was lower in the SEROCO cohort than in HIV controllers (4.3% vs. 80.3%, p<0.001).

**Table 1:** Comparison of baseline characteristics between HIV controllers and patients enrolled in the ANRS SEROCO Cohort and still alive in 2007.

Characteristics	SEROCO Cohort StudyN = 528	HIV Controllers StudyN = 81	p
Women	168 (32.5)	35 (43.2)	0.06
Sexual pref. among men	(n = 357)	(n = 46)	
Heterosexual	163 (45.7)	34 (73.9)	
Homosexual	194 (54.3)	12 (26.1)	<0.001
Caucasians	496 (93.9)	68 (83.8)	
SSA/C*	21 (4.0)	13 (16.2)	<0.001
Others	11 (2.1)	0 (0.0)	
Transmission group			
Sexual	342 (64.8)	42 (51.9)	
IVDU	40 (7.6)	25 (30.9)	<0.001
Others	146 (27.6)	14 (17.2)	
Year of HIV diagnosis	1989 [1988–1990]	1989 [1987–1994]	0.003
Age at HIV diagnosis	27 [23–34]	29 [24]–[32]	0.27
First CD4**/mm^3^	520 [357–682]	795 [627–1000]	<0.001
First viral load** (% < threshold)	20 (4.3)	65 (80.3)	<0.001

Data are median [IQR] or n (%);

*SSA/C  =  SubSaharan Africans or Caribbeans;

**Following HIV diagnosis.

As expected, the HLA-B57 and B27 alleles were over-represented among HIV controllers (respectively 45% and 15% of Caucasian controllers) when compared to the French Caucasian general population (6.2% and 6.9%, respectively; www.allelefrequencies.net).

### Viral load and CD4+ T cell counts in HIV controllers

All available HIV-RNA and CD4 T cell values since HIV diagnosis were collected. The detection limits of viral load assays improved over time: in 1996-99, 36.8% of viral loads were measured with assays having detection limits below 50 copies/ml, compared to 83.0% in 2000–2004 and 95.9% after 2005. As the median year of HIV diagnosis was 1989, the first viral load measurement in the HIV controllers took place a median of 6.9 years [IQR: 3.5–10.0] after HIV diagnosis. Among these first measurements, 80.3% were below the detection limit (range of the detection limits: 20 to 10 000 copies/ml). Viral load was detectable in the first measurement in 16 patients but the levels were low (median 303 copies/mL; range 84 to 2200 copies/mL), and the time to first undetectable VL was rather short: the median time between HIV diagnosis and the first sample with “undetectable” viral load in these 16 patients was only 17.7 [9.2–38.6] months. Four of these 16 patients were known to have had a negative Elisa HIV screening test (the median interval between the last negative serology and the first positive serology was 9.3 [6.2–18.0] months).

The first CD4 T cell count was recorded a median of 2.1 years [IQR: 0.1–5.9] after HIV diagnosis and the median value was 795/mm^3^ [IQR: 627–1000]. The median of all CD4 T cell counts recorded since HIV diagnosis was 777/mm^3^ [IQR: 599–980]; 14% of values were <500/mm^3^, 3% were <350/mm^3^ and 4 were <200/mm^3^ (once in one patient, and three non consecutive assays in another patient).

At enrollment in the HIV controllers study the median CD4 cell count was 741/mm^3^ [IQR: 521–937]; it was below 500/mm^3^ in 22% of patients and below 350 in 5%; VL was below 400 copies/ml in 96.3% of patients and below the detection limit of 50 copies/mL in 74.1% of patients. Among patients who were enrolled with viral load above the detection limit (n = 22), the median VL was 132 copies/mL [range: 43–1443]. Cellular HIV DNA was quantified in 53 patients at enrolment, and the median value was 1.74 log copies/million PBMC [IQR: 1.36–2.07]. The virus was subtype B in 93% of cases.

### CD4 cell kinetics since HIV diagnosis

The patients were divided into three groups on the basis of their viral load history since HIV diagnosis, as follows: patients who were always below the detection limit (n = 30; “no blip” group), patients in whom fewer than 50% of VL values were above the detection limit (n = 39; “rare blip” group), and patients in whom more than 50% of VL values were above the detection limit (n = 12; “frequent blip” group). The mean number of VL measurements/year since 1^st^ VL measurement was similar in the three groups (table 2); the mean delay between two VL measurements was also similar: 7.3 months, 6.7 months and 7.2 months in the no, rare and frequent groups, respectively, p = 0.48. The proportion of assays with a detection limit of ≤50 copies/mL was not lower in the no blip group (74.7%) than in the rare and frequent blip groups (69.8% and 80.3% respectively). In the rare and frequent blip groups only 15.0% and 12.4% of blips exceeded 400 copies/mL, and the median magnitude of the blips did not differ between these 2 groups (115 copies/mL [range: 25–52000] and 152 copies/mL [range: 30–7500] respectively, p = 0.32). The median interval between the first viral load measurement and the first blip was 30.5 months [IQR: 3.7–61.2] in the rare blip group and 4.3 months [IQR: 0.0–19.9] in the frequent blip group (p = 0.007). Clusters of blips were, by definition, more frequent in the frequent group than in the rare blip group. However, most of the blips were transient, i.e., they were followed by a VL below the detection level.

**Table 2:** Main characteristics of HIV controllers according to blip status during follow-up.

Characteristics	No BlipsN = 30	Rare BlipsN = 39	Frequent BlipsN = 12	p
Women	15 (50.0)	14 (35.9)	6 (50.0)	0.44
Caucasians	23 (76.7)	34 (87.2)	11 (91.7)	0.45
Heterosexual preference	26 (86.7)	31 (79.5)	12 (100.0)	0.25
MSM among men (n = 46)	4/15 (26.7)	8/25 (32.0)	0/6 (0.0)	0.38
Intravenous drug users	11 (36.7)	11 (28.2)	3 (25.0)	0.76
Year of diagnosis	1991 [1988–1994]	1989 [1987–1994]	1988 [1987–1989]	0.08
Year of inclusion	2007 [2006–2007]	2006 [2006–2007]	2006 [2006–2007]	0.09
Age at diagnosis, years	28 [26]–[32]	29 [23–33]	28 [24–35]	0.89
Age at inclusion, years	45 [40–49]	45 [39–49]	48 [42–50]	0.56
Number of HIV-RNA measurements	14 [9]–[19]	17 [14]–[22]	17 [11]–[19]	0.09
Number of HIV-RNA measurements/year since 1^st^ VL (mean(std))	1.8 (0.8)	1.9 (0.6)	1.9 (0.6)	0.81
Number of blips (HIV-RNA >detection limit*)	0 [0-0]	4 [2]–[6]	11 [9]–[14]	<0.001
% of blips (HIV-RNA >detection limit)	0.0 [0.0–0.0]	23.5 [14.3–29.4]	75.0 [67.9–83.3]	<0.001
% blips >400 copies/mL	0.0 [0.0–0.0]	15.0 [0.09–0.21]	12.4 [0.07–0.18]	0.53
***At first measurement*** **		14 (35.9)		
First CD4 measurement,/mm^3^	2.8 [0.3	1.3 [0.01	3.0 [0.1	0.16
Time from HIV diagnosis to first HIV-RNA measurement, years	813 [610	795 [641	782 [624	0.73
Time from HIV diagnosis to first CD4 measurement, years	5.9 [3.0	7.8 [2.9	9.7 [7.4	0.11
***At inclusion in the HIV controllers study***				
Time since HIV diagnosis, years	15.1 [11.9–19.1]	16.9 [13.9–19.1]	18.5 [17.2–20.3]	0.13
Time since 1^st^ viral load, years	8.7 [7.0–9.8]	9.7 [8.8–10.7]	9.5 [8.6–9.9]	0.03
CD4 T cell counts,/mm^3^	819 [668–992]	644 [445–890]	628 [569–906]	0.02
HIV-DNA, log_10_ copies/million PBMC	1.78 [1.34–2.18]	1.70 [1.45–2.00]	1.90 [1.62–2.09]	0.73
HIV-RNA >400 copies/mL	0 (0.0)	1 (2.7)	2 (18.3)	0.06
Cancer during follow-up	0 (0.0)	3 (7.7)	1 (8.3)	0.26

Data are median [IQR], mean (std) or n (%);

*whatever the precise detection limit;

**First measurement available after HIV-1 diagnosis.

Table 2 describes the main characteristics of these three subgroups. No difference was found in terms of sex, age or the first CD4 cell count following HIV diagnosis. However, at enrolment in this study the median CD4 T cell count was significantly higher in the no blip group (p = 0.02) than in the other two groups, while the median HIV-DNA level was similar in the three groups.

**Figure 1:**
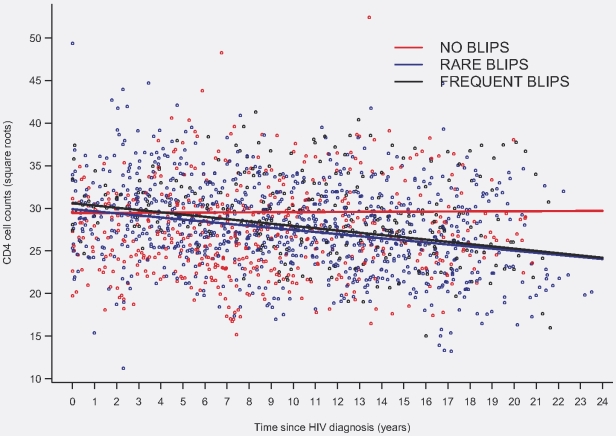
Predicted trajectories of mean √CD4 cell counts since HIV diagnosis according to blip status during the period of HIV control (81 HIV controllers, 1668 CD4 measurements).

In order to better analyze the CD4 cell count kinetics, we estimated the slopes of square-root CD4 T-cell counts by using a mixed-effects linear model. In the no blip group (30 patients), 508 values were used to estimate the slope, with an average of 16 points [range: 6–34] per patient. The corresponding figures were 885 values and 23 points per patient (range: 16–40) in the rare blip group, and 275 values and 22 points per patient (range: 5–47) in the frequent blip group. Overall, the estimated intercept (i.e. the estimated mean CD4 cell count at HIV diagnosis) was 30√CD4/mm^3^ (i.e. 900 CD4) and the slope was -0.17√CD4/mm^3^/year. In other words, during the first year of infection, the CD4 cell count in an HIV controller with 900 CD4 cells/ml at HIV diagnosis fell to 890 CD4/mm^3^ [95%CI: 885.0–899.4], representing a mean decline of 10 CD4/mm^3^. The mean intercept did not differ across the three groups: it was 29.5√CD4/mm^3^ [95%CI: 27.4–31.5] in the no blip group, 29.9√CD4/mm^3^ [25.2–34.6] in the rare blip group and 30.6√CD4/mm^3^ [27.8–36.4] in the frequent blip group. The slope in the no blip group did not differ from zero: +0.01√CD4/mm^3^ per year [95%CI: −0.13; +0.15] (p = 0.89). In contrast, CD4 cell counts in the rare and frequent blip groups fell significantly over time: respectively −0.25 √CD4/mm^3^ per year [95%CI: −0.08; −0.43; p = 0.005], and −0.28 √CD4/mm^3^ per year [95%CI: −0.04; −0.51; p = 0.02]. These two slopes differed significantly from that of the no blip group, but not from each other. Fig. 1 shows the predicted trajectories of mean √CD4 cell counts in the three groups. The results were similar in a more global model that included sex, ethnicity, age at diagnosis (dichotomized around the median of 29 years) and interaction terms between the time since HIV-1 diagnosis and the group. As the rare and frequent blip groups had similar intercepts and slopes, we estimated the slope in all patients with blips: −0.26√CD4/mm^3^ per year [95%CI: −0.09; −0.43; p = 0.003]. This corresponds to a fall in the CD4 cell count from 903 CD4/mm^3^ [95% CI: 818–992] at HIV diagnosis to 830 CD4/mm^3^ [762–901] five years later, 760 CD4/mm^3^ [700–822] 10 years later, and 629 CD4/mm^3^ [558–704] 20 years later.

**Figure 2:**
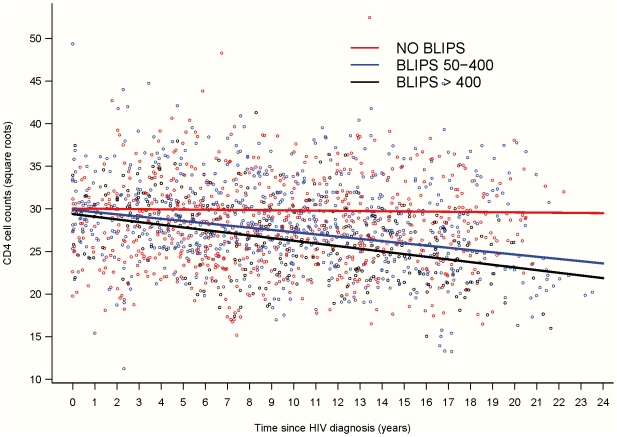
Predicted trajectories of mean √CD4 cell counts since HIV diagnosis according to blip status in the last five years of control (always ≤50 copies vs. blips within 50–400 and blips >400 copies/mL) (81 HIV controllers, 1668 CD4 measurements).

We also conducted a supplementary analysis by restricting the definition of blips to those occurring in the last five years, i.e. when all the patients had their VL measured with an assay with a cut-off for detection ≤50 copies/mL. We could therefore define the no blips group more homogenously by patients having always in the last five years of their follow-up their VL below 50 copies. We found that CD4 behaved differently in patients with viral load always ≤50 copies/mL (no blips group) from patients with blips >50 copies. CD4 slopes in patients with blips within 50–400 copies and patients with blips >400 copies did not differ significantly (figure 2).

### Post-enrolment outcomes

After inclusion in the HIV controllers study, 3 patients experienced immunological HIV disease progression, consisting of a strong and sustained decrease in the CD4 cell count to below 200/mm^3^; one of these patients also had a sustained increase in viral load above 1000 copies/mL. Four patients, including one of the patients in whom the CD4 cell count fell below 200/mm^3^, developed cancer (B cell lymphoma, lung adenocarcinoma, non-differentiated hepatic carcinoma, and gastrointestinal malignancy), and two patients died, one from cardiovascular disease and one from an unknown cause. All three controllers who experienced immunological progression after enrolment belonged to one of the two blip groups, as did the four patients with cancer. The last CD4 cell counts before cancer diagnosis were 225, 521, 977 and 680/mm^3^. No immunological or virological escape or clinical progression was observed in the no blip group.

## Discussion

Spontaneous viral control shortly after HIV-1 seroconversion is not uncommon. Among 330 seroconverters from the ANRS SEROCO cohort, we previously found that 4% of patients had HIV-RNA <400 copies/mL during the 6–24 months following infection, while only one patient had HIV-RNA <40 copies/mL [19]. In the European CASCADE collaboration, spontaneous virological control, defined by at least two consecutive viral loads below the detection limit (<400/500 copies/mL), occurred in 7% of recently infected patients [11]. Long-lasting spontaneous control of viremia is far more rare: here, among 34 317 HIV-seropositive patients managed in 2008 in the 35 French clinical sites participating in the HIV Controllers Observatory, we found that 0.31% of subjects were HIV controllers, a figure close to the 0.5% described in other studies (13, 20).

The factors responsible for HIV control are unclear [4], [21]. As generally reported, we observed a higher proportion of women than men among HIV controllers. However, women were no longer significantly over-represented after adjustment for ethnicity (Africans vs others). In a recent study [15], 100% of natural viral suppressors were African-Americans, who represented 85% of the entire cohort (n = 2484). Our findings suggest that any influence of gender on sustained HIV control might be partly explained by ethnicity.

Relative to the French ANRS SEROCO cohort, homosexual men were under-represented among male HIV controllers in our study, while intravenous drug users were over-represented. Grabar et al. obtained similar results when comparing HIV controllers with other untreated patients [20]. There is no clear explanation for this finding but it is conceivable that homosexual men, as a group, may be treated earlier than drug users, making them ineligible for identification as HIV controllers. It is striking to note that median year of HIV diagnosis in these HIV controllers was 1988 in intravenous drug users and 1992 in homosexual men. We cannot either exclude that the route of infection might play a role. Finally, we also confirm here that HIV controllers have very low levels of cellular HIV-DNA [21].

In the general population of HIV-1-infected patients, the CD4 T cell count declines by 60 to >100 cells/mm^3^ per year [12], [22]–[25]. In a population of long-term nonprogressors (LTNP), the estimated CD4 T cell count decline was 32/mm^3^ per year in patients aged less than 25 years at HIV diagnosis and 16 cells/mm^3^ per year in older patients [10]. In our population of HIV controllers the CD4 cell count decline was much slower, at an estimated 10 cells/mm^3^ per year. The group with highly sustained viral control showed no CD4 T cell decline, whereas the groups with blips had a slight but significant decline over time (approximately 15 CD4/mm^3^ during the first year of HIV infection). This confirms, over a much longer period, findings from a recent study [26] in which the slope of the CD4 cell count differed between controllers with VL <1 copy/mL and those with persistent low-level viremia ≥1 copy/mL. In another study we found that 19 HIV controllers from the Observatory fell into two groups with respect to the HIV-suppressive capacity of their CD8 T cells and their HIV-specific CD8 T cell numbers: 14 patients had a high frequency of HIV-specific CD8 T cells with marked and stable HIV-suppressive capacity and higher levels of T cell activation, while the other five patients had very weak HIV-specific responses and immune activation [27]. These latter five patients with weak responses had experienced no VL blips, while 11 of the 14 patients with strong CD8+ T cell responses had had blips. Thus, in keeping with recent data [28], [29], low-level viremia might be important for sustaining an effective CD8+ T cell response, but the price might be a faster CD4+ T cell decline and an increased risk of virologic failure and clinical events. Interestingly, the three controllers who experienced CD4 T cell declines below 200/mm^3^ and the four patients who developed cancer had all experienced blips. Malignancies in blip patients might be related to chronic immune activation and/or inflammation [30]. Thus, HIV controllers who experienced viral blips and/or chronic immune activation [31], [32], might be more at risk of malignancies and thus require closer follow-up. We are currently implementing a prospective cohort of HIV controllers designed to determine the long-term prognosis, including changes in the CD4 cell count and viral load, and the risk of cancer.
